# Differential impact of lipopolysaccharide defects caused by loss of RfaH in *Yersinia pseudotuberculosis* and *Yersinia pestis*

**DOI:** 10.1038/s41598-017-11334-6

**Published:** 2017-09-07

**Authors:** Jared M. Hoffman, Shea Sullivan, Erin Wu, Eric Wilson, David L. Erickson

**Affiliations:** 1 Department of Microbiology and Molecular Biology, 4007 LSB, Brigham Young University, Provo, UT 84602 USA; 2 Utah Public Health Laboratory, 4431 South 2700 West, Taylorsville, UT 84129 USA

## Abstract

RfaH enhances transcription of a select group of operons controlling bacterial surface features such as lipopolysaccharide (LPS). Previous studies have suggested that *rfaH* may be required for *Yersinia pseudotuberculosis* resistance to antimicrobial chemokines and survival during mouse infections. In order to further investigate the role of RfaH in LPS synthesis, resistance to host defense peptides, and virulence of *Yersinia*, we constructed Δ*rfaH* mutants of *Y*. *pseudotuberculosis* IP32953 and *Y*. *pestis* KIM6+. Loss of *rfaH* affected LPS synthesis in both species, resulting in a shorter core oligosaccharide. Susceptibility to polymyxin and the antimicrobial chemokine CCL28 was increased by loss of *rfaH* in *Y*. *pseudotuberculosis* but not in *Y*. *pestis*. Transcription of genes in the *ddhD-wzz* O-antigen gene cluster, but not core oligosaccharide genes, was reduced in Δ*rfaH* mutants. In addition, mutants with disruptions in specific *ddhD-wzz* O-antigen cluster genes produced LPS that was indistinguishable from the Δ*rfaH* mutant. This suggests that both *Y*. *pseudotuberculosis* and *Y*. *pestis* produce an oligosaccharide core with a single O-antigen unit attached in an RfaH-dependent fashion. Despite enhanced sensitivity to host defense peptides, the *Y*. *pseudotuberculosis* Δ*rfaH* strain was not attenuated in mice, suggesting that *rfaH* is not required for acute infection.

## Introduction

*Yersinia pestis* is a recently emerged clone of *Y*. *pseudotuberculosis*, and these two species provide a fascinating model for investigating the evolution of bacterial pathogens. *Y*. *pestis* is transmitted via an infected flea bite and is the causative agent of plague^1^. *Y*. *pseudotuberculosis* (*Yptb*) is a zoonotic pathogen, typically acquired by ingestion of contaminated food or water, that causes self-limiting gastroenteritis in humans. Both species share a tropism for growth in lymph nodes^2^.

*Yersinia* survival and replication within the small intestine, Peyer’s patches, liver, and spleen is enhanced by the carriage of a 70 kb virulence plasmid called pYV (or pCD1 in *Y*. *pestis*)^3^. This plasmid encodes a type III secretion system that injects *Yersinia* outer proteins (Yops)^4^. Yops have a wide variety of functions including counteracting pro-inflammatory cytokine production and preventing phagocytosis^5, 6^. However, *Yptb* P^−^ mutants lacking the pYV plasmid grow equally well in the mesenteric lymph nodes following oral infection^7^. In an effort to understand how P- strains survive in the absence of a type III secretion system, Crimmins *et al*. conducted a genome-wide screen to identify putative chromosomal virulence factors that enable survival in lymphoid tissues. Several mutants that appeared to have colonization defects had insertions in genes involved in lipopolysaccharide (LPS) synthesis, including *rfaH* ^7^.

RfaH was originally identified as a component in the synthesis of LPS of *Salmonella enterica* serovar *typhimurium* ^8^. Since this initial discovery, RfaH has been implicated in a wide array of processes in gammaproteobacteria, including F-plasmid conjugation^9^, hemolysin toxin production^10^, and expression of type II K15 capsule^11^. RfaH functions in a similar fashion to the essential NusG protein. It allows the RNA polymerase to bypass intrinsic terminator sites or DNA binding proteins in order to completely transcribe long operons^12–14^. The specificity of RfaH to its target genes depends upon a conserved regulatory site called the operon polarity suppressor (*ops*)^15^, which is typically found within the 5′-proximal transcribed sequence of operons regulated by RfaH. In *E*. *coli rfaH* mutants, genes proximal to the promoter of RfaH-regulated operons are moderately repressed, but the transcription of more distal genes is more reduced, resulting in transcriptional polarity^16^. The activity of RfaH in *E*. *coli* can also be inhibited by the small RNA RirA, leading to LPS defects and activation of the RpoE stress response^17^.

In *Yptb* and *Y*. *pestis*, the *ddhD-wzz* O-antigen gene cluster possesses a canonical *ops* element upstream of the *ddhD* gene and is thus a likely target for RfaH regulation. We recently identified *Yptb* transposon mutants with altered resistance to antimicrobial chemokines CCL25 and CCL28^18^. While alterations to the oligosaccharide core had a strong effect on antimicrobial chemokine resistance, transposon mutants in the *rfaH* gene as well as mutants within the *ddhD-wzz* serotype O:1b O-antigen cluster were also identified in this screen. Previously, Karlyshev *et al*.^19^ and Mecsas *et al*.^20^ found that transposon insertions in genes predicted to be required for serotype O:3 O-antigen synthesis compromised the ability of *Yptb* to colonize mouse organs following orogastric, intraperitoneal, or intravenous injection.

An important question that arises from these earlier studies is whether defects in mouse colonization and host defense peptide resistance that have been ascribed to *Yptb rfaH* mutations are due to lack of O-antigen synthesis or to alterations in the core oligosaccharide. Loss of RfaH in *Y*. *enterocolitica* serotype O:3 reduces expression of both O-antigen and outer core gene clusters of the LPS^21^, and putative *ops* sites were identified proximal to both O-antigen and outer core gene clusters in *Y*. *enterocolitica*. However, the structure and genetic organization of these regions in *Yptb* and *Y*. *pestis* is significantly different from *Y*. *enterocolitica*. Furthermore, since *Y*. *pestis* fails to produce O-antigen, the role of RfaH in this species is difficult to predict.

In this study, we sought to determine the role that the *rfaH* gene plays in the synthesis of the O-antigen and core oligosaccharide in *Yptb* serotype O:1b strain IP32953 and in *Y*. *pestis* strain KIM6+. We examined whether loss of *rfaH* affects gene expression and resistance to host defense peptides in both species. Additionally, *Yptb* survival in mouse organs in both P^+^ and P^−^ virulence plasmid strain backgrounds was determined. Results suggest that RfaH controls the addition of a single O-antigen unit to the LPS core, thereby increasing resistance to antimicrobial chemokine CCL28 and polymyxin in *Yptb*. Despite greater sensitivity to host defense peptides, loss of *rfaH* did not affect bacterial survival *in vivo*.

## Results

### Effect of RfaH on lipopolysaccharide synthesis in *Y*. *pseudotuberculosis* and *Y*. *pestis*

To determine the role of the *rfaH* gene in *Yptb* and *Y*. *pestis*, the entire coding region was first deleted from *Y*. *pestis* using lambda-red recombination. After the mutation was made in *Y*. *pestis*, the modified allele (*rfaH* upstream and downstream regions flanking a kanamycin resistance gene) was transferred to *Yptb* by allelic exchange. After verifying the genotypes of the resulting mutants (Supplementary Fig. S1), we determined whether RfaH influences the synthesis of LPS in these species.

**Figure 1:**
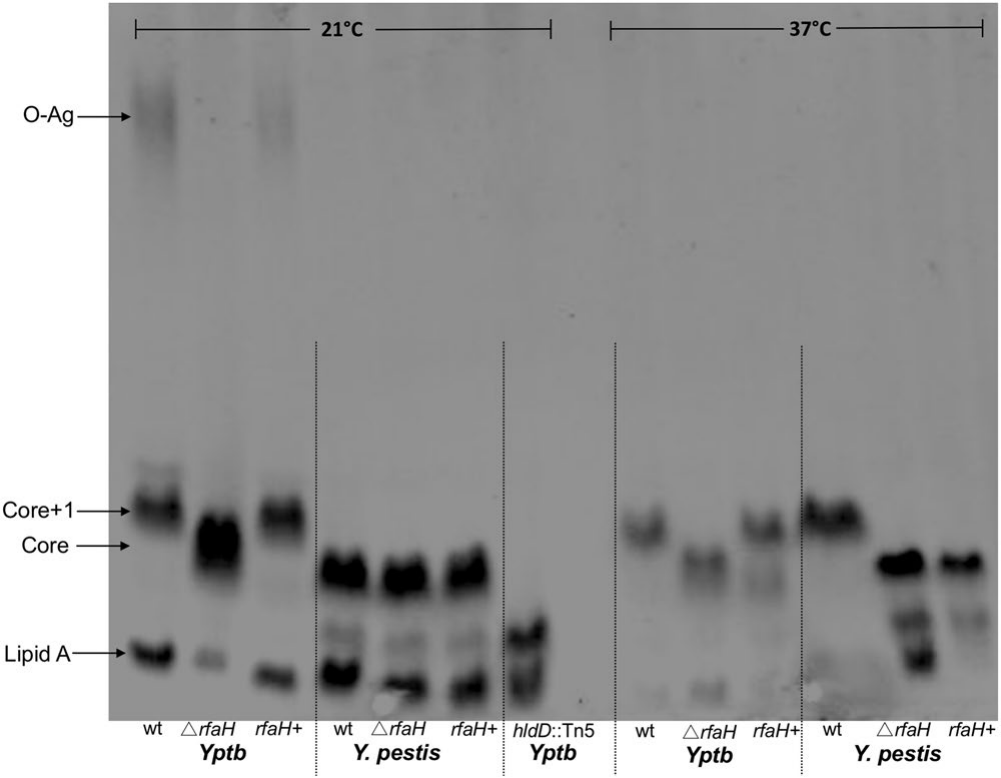
LPS changes resulting from loss of *rfaH* in *Yptb* and *Y*. *pestis*. LPS fractions were isolated from the indicated strains grown at either 21 or 37 °C, separated by SDS-PAGE, and stained. In the *Yptb* Δ*rfaH* mutant the high molecular weight O-antigen and the larger (core+1OPS) oligosaccharide are reduced compared to the wild type. These phenotypes are complemented with the *rfaH*+ plasmid in *Yptb*. In *Y*. *pestis*, the wild type strain produces larger core+1OPS oligosaccharide at 37, but the Δ*rfaH* mutant strain does not. The *rfaH*+ plasmid fails to complement the Δ*rfaH Y*. *pestis* mutant.

**Figure 2:**
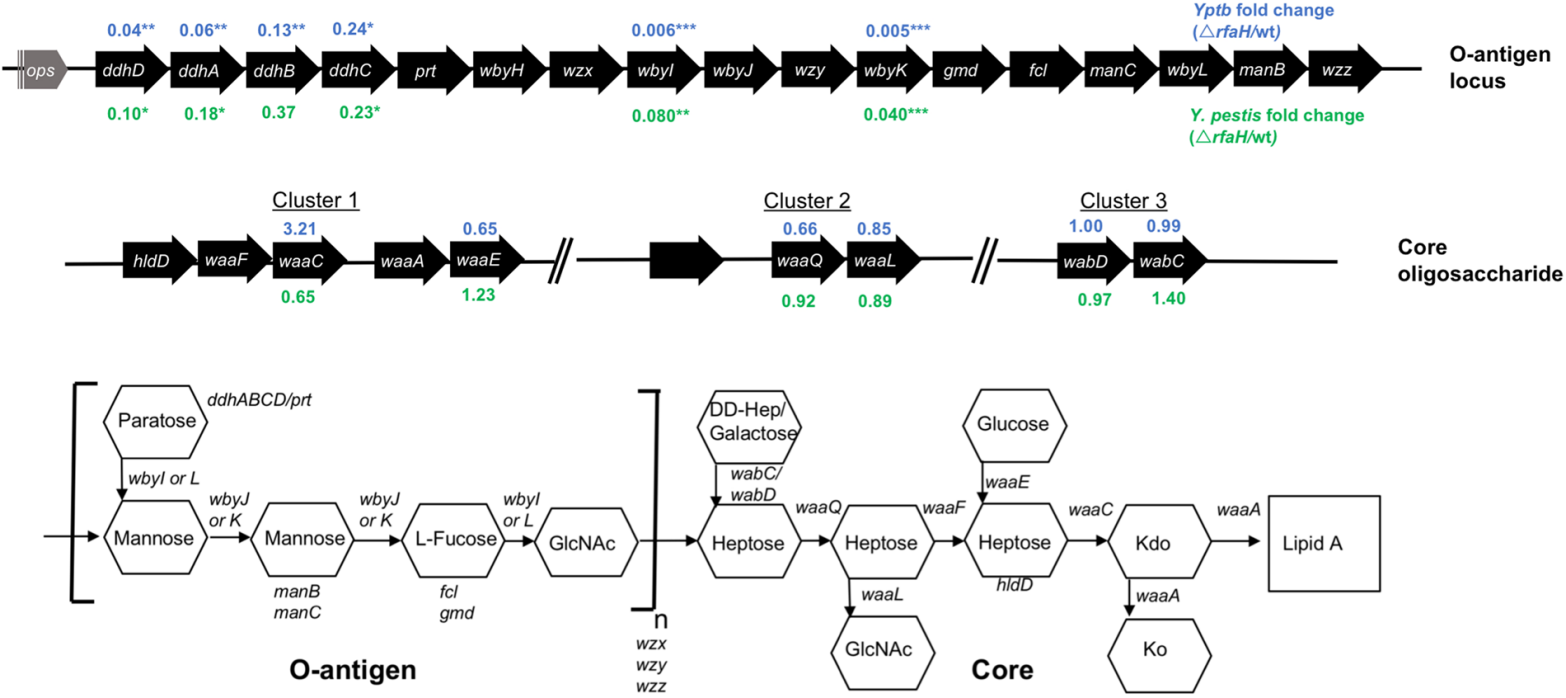
Transcriptional changes in LPS genes caused by loss of *rfaH*. The *ddhD-wzz* O-antigen gene cluster (top) contains an *ops* regulatory sequence proximal to *ddhD*. The core oligosaccharide genes are found in 3 clusters as indicated (middle) and do not have *ops* sequences associated with them. The expression of individual LPS genes in the Δ*rfaH* mutant compared to the wild type strains of *Yptb* (blue) or *Y*. *pestis* (green) were measured by qPCR. Data represent the mean fold changes in expression of specific genes, and statistically significant differences are indicated (*p < 0.05, **p < 0.01, ***p < 0.001). Similar results were obtained in two independent experiments, and data shown are from one representative experiment done in triplicate. The *ddhD-wzz* O-antigen genes are regulated by *rfaH* whereas the core oligosaccharide genes are not. The bottom image depicts the function of individual genes in producing *Yptb*/*Y*. *pestis* LPS.

In *Yptb and Y*. *pestis* the composition of the sugars in LPS is known to change depending on growth temperature. We therefore extracted LPS from cultures grown at 21 °C and 37 °C from wild-type and mutant strains and analyzed these via polyacrylamide gel electrophoresis and staining of carbohydrates (Fig. 1). As expected, based on the *ops* element upstream of the O-antigen *ddhD* gene (Fig. 2), the *Yptb* Δ*rfaH* strain produced less high molecular weight O-antigen at 21 °C. Neither the wild-type or the *Yptb* Δ*rfaH* mutant strain produced O-antigen at 37 °C, which is consistent with known temperature-dependent O-antigen regulation in this strain. Restoration of O-antigen production in the *Yptb* Δ*rfaH* mutant was achieved by inserting the *rfaH* gene on plasmid pACYC184, which exists at about 15 copies per cell.

In contrast to the single O-antigen biosynthetic cluster, the core oligosaccharide genes exist in three separate clusters in *Yptb* and *Y. pestis* (Fig. 2). Cluster 1 contains the genes required for inner core synthesis including *hldD*, *waaF* and *waaC*. The outer core genes are contained in Clusters 2 (*waaL* and *waaQ*) and Cluster 3 (*wabD* and *wabC*). No *ops*-like sequences are apparent anywhere in Clusters 1–3, which suggests that RfaH likely does not play a role in transcriptional regulation of these operons. Nevertheless, we found that deleting *rfaH* altered the size of the *Yptb* oligosaccharide core at 21 and both species at 37 °C (Fig. 1). The *rfaH*+ plasmid successfully complemented O-antigen production in the *Yptb* Δ*rfaH* mutant, but did not affect size of the core oligosaccharide in the *Y*. *pestis* Δ*rfaH* mutant. As a size comparison, we included LPS from a *Yptb hldD*::Tn5 mutant strain, which fails to add l–d Heptose to the inner core. The Δ*rfaH* mutant core was significantly larger than that of *hldD*::Tn5 core, indicating that reduced transcription of the inner core was not likely responsible for the observed size change. We therefore considered that this size difference could reflect an alteration to the outer core or that conversely, *Yptb* (at 21 °C and 37 °C) and *Y*. *pestis* (at 37 °C) may produce a core with a small number of O-antigen oligosaccharide units (core+1OPS) in an RfaH-dependent process.

The LPS size changes we observed in the strains lacking *rfaH* (Fig. 1) suggested that expression of O-antigen and possibly outer core oligosaccharide biosynthesis genes may be regulated by RfaH. To test between these possibilities, RNA was isolated from wild-type and Δ*rfaH* mutant bacteria of both *Yptb* and *Y*. *pestis*. Transcription of several O-antigen and core oligosaccharide synthesis genes was measured using quantitative real-time PCR. As shown in Fig. 2, transcription of the *ddhD* gene cluster was significantly downregulated in the Δ*rfaH* mutant strains. Consistent with function of RfaH as an antiterminator, the downregulation of the more distal genes (*wbyI* and *wbyK*) was more pronounced than those closer to the promoter (*ddhD* and *ddhA*). Additionally, the effect of the Δ*rfaH* mutation was more pronounced in *Yptb* than in *Y*. *pestis*. Quantitative analysis of the transcription of core synthesis genes in clusters 1–3 showed that they were not significantly different in the Δ*rfaH* mutants, which is consistent with the absence of an *ops* sequence in these regions. These gene expression results suggested that the truncation we observed in the LPS of the Δ*rfaH* mutants was due to downregulation of the *ddhD-wzz* O-antigen cluster and not core oligosaccharide gene transcription.

**Figure 3:**
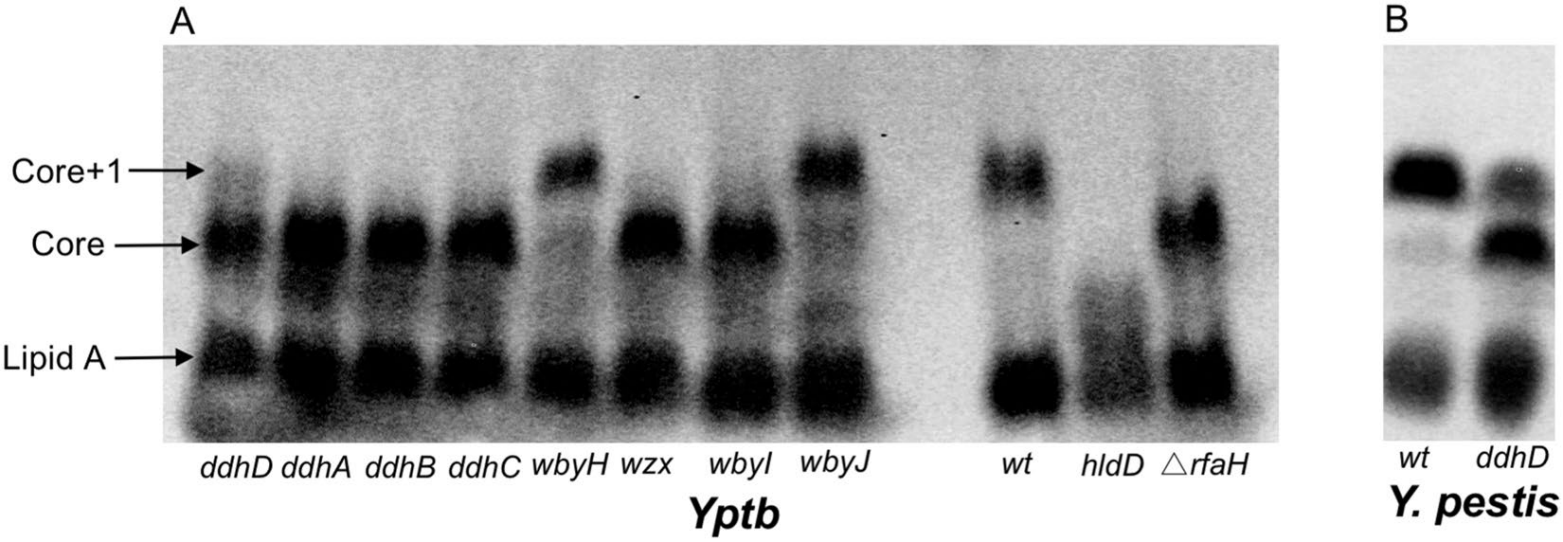
Individual *ddhD-wzz* O-antigen cluster genes are needed for production of core +1OPS in *Yptb* (**A**) and *Y*. *pestis* (**B**). LPS was isolated from the wild type strains, specific Tn5 insertion mutants mapped to individual genes as indicated, or the Δ*rfaH* mutant and separated by SDS-PAGE.

We next hypothesized that if the altered LPS size in the Δ*rfaH* mutants were caused solely by reduced transcription of the *ddhD-wzz* cluster, then mutations in this cluster would also produce alterations in the LPS migration pattern resembling the Δ*rfaH* mutant strain. LPS from a series of *Yptb* Tn5 transposon mutants^22^ that mapped to individual genes in this cluster supported this interpretation (Fig. 3A). The sizes of the LPS fractions for the mutants with insertions in *ddhDABC*, *wzx*, and *wbyI* genes were indistinguishable from the Δ*rfaH* mutant. The *wbyH* and *wbyJ* (putative glycosyltransferase) Tn5 mutants produced LPS similar in size to the wild type strain, suggesting that these two genes are not essential for O-antigen synthesis. We also analyzed LPS from a *Y*. *pestis ddhD*::Tn5 mutant (Fig. 3B). Similar to the mutation in *Yptb*, disruption of the *ddhD* gene in *Y*. *pestis* strongly reduced production of the larger putative core+1OPS oligosaccharide in comparison to the wild type strain (Fig. 3B).

### RfaH is necessary for protection against CCL28 and polymyxin in *Yptb* but not *Y*. *pestis*

**Figure 4:**
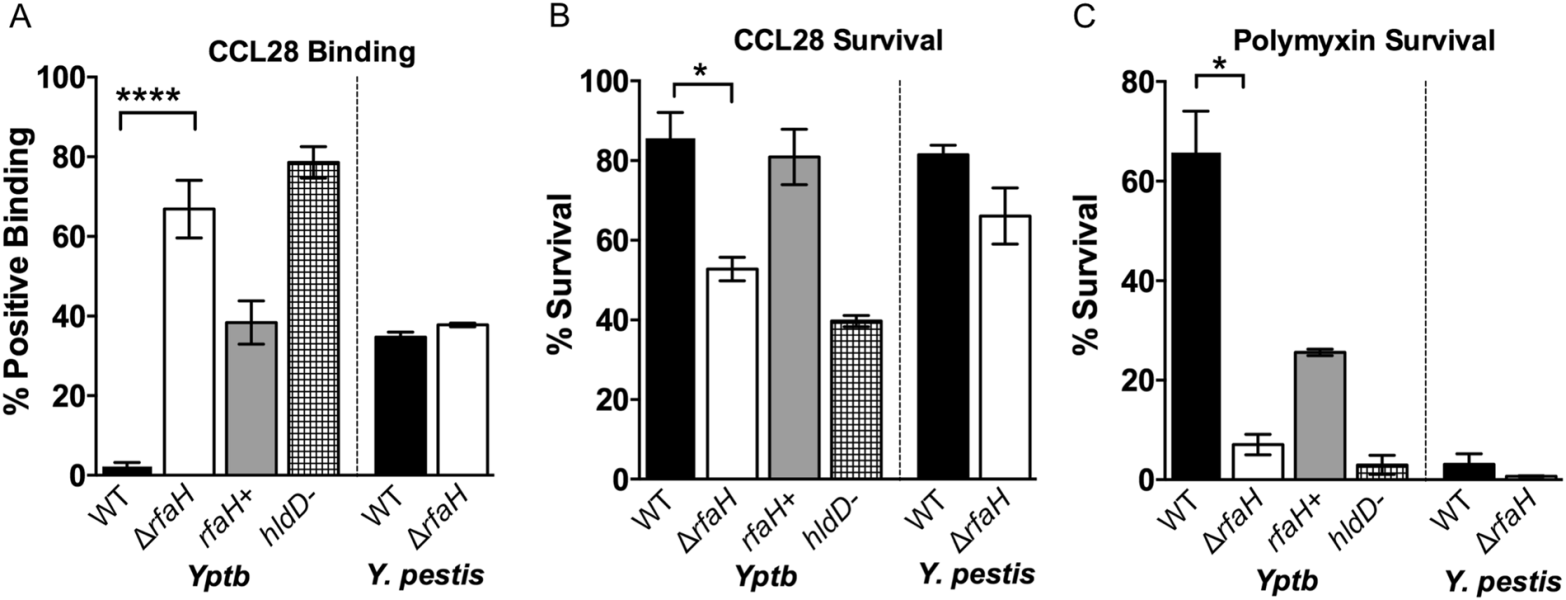
*Yptb rfaH* affects antimicrobial peptide susceptibility but *Y*. *pestis rfaH* does not. (**A**) Binding of CCL28 to *Yptb* but not *Y*. *pestis* is significantly enhanced by loss of *rfaH*, expressed as a percentage of cells that stain positive by flow cytometry. (**B** and **C**) Relative survival of bacteria (expressed as a percentage of the number of live cells counted in the unexposed control of the same strain) in the presence CCL28 (**B**) or polymyxin (**C**). Asterisks denote that the result obtained was significantly different from the wild type strain at the given concentration by Two-way ANOVA (****p < 0.0001, *p < 0.05). Similar results were obtained in three independent experiments, and data shown are from one representative experiment done in triplicate.

As LPS plays an important role in the defense against host antimicrobial peptides, *Yptb* and *Y*. *pestis* strains were examined to determine whether the loss of *rfaH* decreased resistance of the bacteria to the antimicrobial chemokine CCL28 and to polymyxin. We first measured the impact of *rfaH* deletion on binding to CCL28 using flow cytometry. As shown in Fig. 4A, deletion of *rfaH* from *Yptb* increased the proportion of cells that bind CCL28 from near zero in the wild type to approximately seventy percent in the mutant strain. Complementation of the mutant with the *rfaH* plasmid significantly reduced binding to CCL28 to near wild type levels. Interestingly, the level of CCL28 binding observed for the Δ*rfaH* mutant was comparable to the *Yptb* *hldD*::Tn5 strain lacking l-d Heptose inner core residues, suggesting that there is a threshold at which increased truncation of LPS does not significantly change access of the antimicrobial peptide to the bacterial surface.

We also measured *Yptb* survival in the presence of CCL28 and polymyxin. Consistent with the binding results, the *Yptb* wild-type strain was largely unaffected by CCL28 whereas the Δ*rfaH* strain exhibited enhanced sensitivity (Fig. 4B). Complementation with the *rfaH*+ plasmid restored the survival rates to wild-type levels. Susceptibility to polymyxin was also affected by *rfaH* mutation. *Yptb* is relatively resistant to polymyxin, but as shown in Fig. 4C, the Δ*rfaH* strain shows a dramatic decrease in survival when exposed to polymyxin as compared to the wild-type. Consistent with the CCL28 survival results, the complemented strain and the wild type strain showed similar resistance to polymyxin.

In contrast to *Yptb*, CCL28 binding did not detectably change as a result of *rfaH* mutation in *Y*. *pestis* (Fig. 4A). We also found that *rfaH* mutation did not affect bacterial survival in the presence of CCL28, with the wild type and mutant strains exhibiting an 80% percent survival rate similar to the *Yptb* wild type strain (Fig. 4B). Interestingly, although *Y*. *pestis* appears similarly susceptible to the chemokine CCL28 as *Yptb* (independently of *rfaH*), *Y*. *pestis* is far more sensitive to polymyxin than *Yptb* at 37 °C (Fig. 4C). This sensitivity was slightly enhanced by loss of *rfaH* in *Y*. *pestis*, but the difference was not statistically significant under these conditions. These results suggest that addition of unpolymerized O-antigen to the *Y*. *pestis* outer core does not significantly affect susceptibility to these antimicrobial peptides. Conversely, the putative core +1OPS contributes to the polymyxin and CCL28 resistance of *Yptb*.

### RfaH does not affect *Yptb* acute virulence following oral and intravenous mouse infections

**Figure 5:**
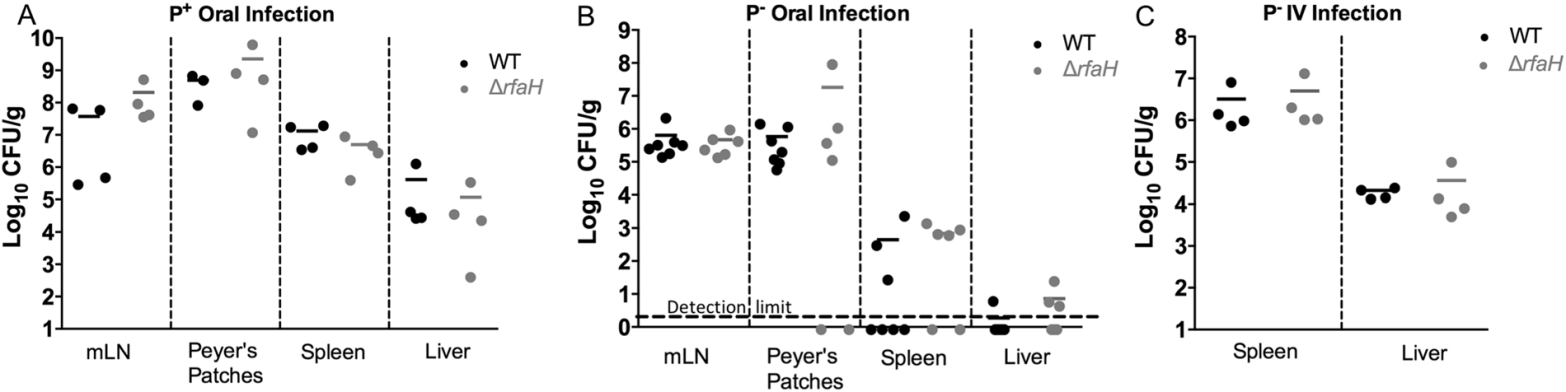
Loss of *rfaH* in *Yptb* does not affect bacterial replication and dissemination. Mice were infected with 10^7^ CFU of IP32953 wild type or Δ*rfaH* mutant containing the pYV virulence plasmid (P^+^) or 10^8^ CFU of the strains without the plasmid (P^−^). (**A**) Scatterplot of bacterial burden in each organ following oral infection with P^+^ strain. (**B**) Scatterplot of bacterial burden following oral infection with P^−^ strain. (**C**) Scatterplot of bacterial burden in spleen and liver following intravenous infection with P^−^ strain. Each dot represents 1 mouse, with the mean indicated. Data represent 3 independent experiments (n = 1–4 per experiment). No significant differences were detected when bacterial burdens for the wild type or Δ*rfaH* mutants in any of the organs were compared (Mann-Whitney test).

The truncated LPS and increased susceptibility to antimicrobial peptides in the *Yptb* Δ*rfaH* strain suggested that this strain would exhibit a survival defect during *in vivo* mouse infections. To test this hypothesis, we first compared the ability of *Yptb* wild-type and Δ*rfaH* strains that carry the pYV virulence plasmid (P^+^) to colonize after oral infection. Three days after infection, the mesenteric lymph nodes, Peyer’s patches, spleen, and liver were collected and the bacterial loads determined. As shown in Fig. 5 there was no significant difference in survival of wild-type P^+^ and Δ*rfaH* P^+^ bacteria at this time point. Mice infected with the wild type strain or the Δ*rfaH* mutant appeared equally sick, with ruffled fur and lethargy prior to being euthanized. Given this unexpected result, we next determined whether *rfaH* would affect *in vivo Yptb* survival in strains lacking the pYV virulence plasmid (P^−^). As observed with the P^+^ infections, there were no significant differences in bacterial numbers in any organ between the P^−^ Δ*rfaH* and wild-type strains (Fig. 5B). These results clearly indicate that *rfaH* is not required for survival and dissemination of *Yptb* strain IP32953 after oral infection in mice three days post infection.

Previous studies have also suggested that transposon insertion mutations in *rfaH* may result in reduced ability to colonize via intravenous infection^7^. To investigate this possibility, we infected groups of mice via intravenous retro-orbital infections with P^−^ *Yptb* wild-type Δ*rfaH* mutant bacteria. The bacterial loads in the liver and spleens of these mice were measured three days following infection (Fig. 5C). Similar to the oral infection results, survival of the Δ*rfaH* mutant was not decreased in comparison to wild-type bacteria in any of the organs. These data demonstrate that although *rfaH* deletion alters LPS structure and increases susceptibility to antimicrobial peptides *in vitro*, these changes do not significantly affect bacterial survival during mouse infection.

## Discussion

As has been seen in other bacterial species, we found that RfaH regulates the synthesis of the LPS in both *Yptb* and *Y*. *pestis*. Loss of *rfaH* eliminated high molecular weight O-antigen production in *Yptb* and caused truncation of a portion of the LPS that, because of its size, we initially suspected was within the core oligosaccharide (Fig. 1). Gene expression analysis indicated that core oligosaccharide genes were not affected; however, several genes within the *ddhD-wzz* O-antigen gene cluster were downregulated in the Δ*rfaH* strains (Fig. 2). In agreement with these gene expression results, LPS from several mutants with disruptions in the *ddhD-wzz* O-antigen locus had the same electrophoretic mobility as LPS from the Δ*rfaH* mutant (Fig. 3). This demonstrates that the *ddhD-wzz* cluster is the relevant target of RfaH responsible for the LPS truncation. Semi-rough LPS consisting of lipid A plus core oligosaccharide with a single O-antigen unit has been observed in *Yptb* O3, O4, and O8 strains^23, 24^. Other *Y*. *enterocolitica* O:3 and O:9 strains also have a gene cluster required for synthesis of a single O unit that is not polymerized but which is attached to the inner core of the LPS^25, 26^. However, no reports of *Y*. *pestis* strains producing semi-rough LPS have been published previously. Our results suggest that both *Yptb* IP32953 (O:1b) and *Y*. *pestis* KIM6+ can produce this form at 37 °C and *Yptb* at both 21 and 37 °C.

It is interesting that although *Y*. *pestis* strains are believed to be rough, all of them retain the *ddhD-wzz* gene cluster. Early sequencing of *Y*. *pestis* strains CO92 and EV76 indicated that several O-antigen genes (eg. *ddhB*, *wbyI*, *gmd*, and *fcl*)^27, 28^ were likely inactivated early in the emergence of *Y*. *pestis* from its ancestral strain. However, examination of more recently added genomes from diverse strains predicts that several carry functional versions of at least some of these genes. It has also been suggested that some of the mutations may be phase-variable since they occur in repetitive sequences prone to mismatch repair^27^. We have shown here that a transposon disruption within *ddhD* changes the LPS electrophoretic mobility in *Y*. *pestis* KIM6+ (Fig. 3B), showing that this locus does have a function in *Y*. *pestis*. This *ddhD*::Tn5 mutant was identified based on altered colony phenotype on Congo-red agar plates and it exhibits increased clumping in liquid media, phenotypes that are consistent with altered cell envelope properties. It is unlikely that any *Y*. *pestis* strains produce high molecular weight polymerized O-antigen as it is known to interfere with the function of the plasminogen activator protease^29, 30^, which is essential for plague pathogenesis^31^. Our studies are the first to our knowledge to suggest the production of oligosaccharide with a single O-unit in *Y*. *pestis*. It is also interesting that this form only appeared during growth at 37 °C. The structure of the core oligosaccharides of some *Y*. *pestis* strains has been determined via high-resolution analyses including NMR and electrospray ionization mass spectrometry (ESI MS)^32, 33^. These investigations did not detect the O-antigen sugars paratose, fucose, or mannose. However, they did find temperature-dependent differences in the oligosaccharide composition. Differences in serologic specificities of antibodies to LPS from *Y*. *pestis* have been suggested, thought to be primarily due to temperature-dependent variations in the structural properties of lipooligosaccharides^34^. Similar detailed structural analysis of LPS from additional *Y*. *pestis* strains may be warranted to verify the effect of RfaH that we propose in these studies.

Changes in LPS can significantly affect resistance of Gram-negative bacteria to complement and antimicrobial peptides. Loss of *rfaH* altered LPS structure in *Yptb*, dramatically increasing susceptibility to polymyxin B (Fig. 4). We observed similar trends with binding and killing by the antimicrobial chemokine CCL28 in the Δ*rfaH* mutant. However, in *Y*. *pestis* we saw no significant difference in polymyxin or CCL28 susceptibility between the wild type and the Δ*rfaH* strains. Additionally, even though *Y*. *pestis* is much more sensitive than *Yptb* to polymyxin, it is equally susceptible to CCL28. This may indicate polymyxin and CCL28 have different targets, or that some features of *Y*. *pestis* not found in *Yptb*, such as capsule or the Pla protease (carried on plasmids pPCP1 and pMT1), could limit the access of CCL28 (but not polymyxin) to the bacterial surface^35^.

Plasmid complementation with the *rfaH* gene mostly restored the wild-type phenotypes in the *Yptb* Δ*rfaH* strain in the LPS analyses and antimicrobial assays. We attempted to complement the *Y*. *pestis* Δ*rfaH* mutant using the same plasmid with the *rfaH* gene from *Yptb*, but surprisingly this plasmid failed to restore the wild-type phenotypes. Since the *Y*. *pestis rfaH* sequence differs from the *Yptb* sequence by one nucleotide (causing a single glycine-valine difference at position 75, see Supplementary Fig. 1), we also created a plasmid containing the *Y*. *pestis* version of this gene. This plasmid also failed to restore the wild-type phenotypes (data not shown).

Cases where phenotypes are unable to be complemented in mutant strains can be due to additional compensatory mutations in non-target genes or disruptions to flanking genes during mutagenesis. PCR reactions with primers within genes flanking *rfaH* (*hemB* and *ubiD*) gave the expected size products (Supplementary Fig. 1), suggesting that the recombination had not disrupted nearby genes. The *Y*. *pestis* Δ*rfaH* mutant was remade using the same allelic exchange plasmid which was used to generate the *Yptb* mutant strain. The same mutant phenotypes were observed in this new strain, but again the mutation was not able to be complemented via transformation with either the *rfaH*_*YPTB*_ or *rfaH*_*pestis*_ plasmids. This result suggests that secondary, non-target mutations may arise extremely quickly in the *Y*. *pestis* Δ*rfaH* strains that prevent restoration of RfaH function. Alternatively, we considered the possibility that in *Y*. *pestis* multiple copies of the *rfaH* gene carried on plasmids could result in incorrect expression levels or other effects that prevent proper function. Therefore, a separate complementation strategy was attempted using a Tn7 transposon to insert a single copy of *rfaH* into the chromosome in the Δ*rfaH* strains. However, despite successful insertion of *rfaH* at the Tn7 site, the LPS remained truncated in this strain (data not shown). After multiple attempts via different methods it remains unclear why complementation of the *rfaH* gene in *Y*. *pestis* has not been successful. Because of the absence of complementation, at this time we cannot rule out the possibility that expression changes observed in the *Y*. *pestis* Δ*rfaH* mutant are influenced by other mutations.

The importance of *rfaH* to virulence of *E*. *coli* and *Salmonella* is well established, and *rfaH* mutants are sufficiently attenuated to make them potential live vaccine candidates^36–38^. Given its potential role in host immune evasion, RfaH could be an attractive target for the development of new anti-virulence treatments against these species. Recent studies have also suggested a possible role for *rfaH* in *Yersinia* pathogenesis. For instance, a *Y*. *enterocolitica* Δ*rfaH* mutant was shown to have greater sensitivity to polymyxin, but more resistance to serum complement^21^. We also previously identified *rfaH* in a screen for *Yptb* IP32953 mutants with increased antimicrobial chemokine binding^18^ suggesting a role for RfaH in bacterial colonization. Other groups have also found that mutants with transposon insertions in *rfaH* in a *Yptb* YPIII P^−^ strain background were less competitive for growth in liver and spleen following intravenous infection in BALB/c mice^7^. Mutation of O-antigen genes, which we show here are regulated by RfaH (Fig. 2), reduced survival in competitive *Yptb* genome-wide transposon mutagenesis studies following orogastric, intraperitoneal, or intravenous infection of mice^19^. These high-throughput screens involving competition between thousands of mutants strongly implicate RfaH and genes regulated by RfaH in virulence. In previous studies, fitness defects observed in *Yptb rfaH* mutants were calculated to be up to 100,000-fold^7^. In addition, during the course of our studies Green *et al*.^39^ demonstrated that in a 1:1 competition with the wild-type strain, *Yptb* strain IP26666 *rfaH* mutants have an approximately 10-fold growth defect in mouse livers and spleens after intravenous injection. They also found that the fitness defect of this mutant was even further enhanced when mice were first depleted of neutrophils.

In this study, single strain infections were performed comparing the virulence of the wild type and an Δ*rfaH* mutant IP32953 serotype O:1b strain. Unexpectedly, we found that the Δ*rfaH* mutant did not appear to be attenuated, regardless of whether the plasmid encoding the type III secretion system was present, or whether the mice were infected orally or intravenously (Fig. 5). In addition to the strain and serotype differences between the strains used in our study and those published previously, it is likely that competition assays would give a more sensitive measure of any defects caused by *rfaH* mutation. It is also possible that measuring bacterial colonization at earlier or later time points could reveal subtle differences between the wild type and mutant strains used here. However, our results suggest that *rfaH* mutation by itself may not be universally sufficient for *Yptb* attenuation, and may lessen the attractiveness of RfaH as an antibacterial target for *Yersinia*.

## Materials and Methods

### Bacterial strains and growth conditions

*Y*. *pestis* KIM6+ and *Yptb* serotype O:1b IP32953 were routinely grown in Terrific Broth (TB) at either 21 °C or 37 °C. Kanamycin (30 µg/mL) and chloramphenicol (10 µg/mL) were included when necessary. *Escherichia coli* strain MFDλpir was grown in Luria Broth (LB) at 37 °C. *Yptb* mutants with Tn5 transposon insertion in *hldD* and in genes within the *ddhD-wzz* locus were previously described^18^. A *Y*. *pestis* mutant with an insertion in *ddhD* was obtained using the same transposon delivery method and selection strategy.

### Gene Deletions and Complementation

The *rfaH* gene was deleted from *Y*. *pestis* via lambda-red recombination^40, 41^. Primers (Supplementary Table S1) were designed to amplify three individual segments with complementary overhangs, representing 500 bp upstream and downstream segments flanking the *rfaH* gene, and the kanamycin resistance gene from plasmid pKD13. These three PCR products were combined using overlap-extension PCR. This DNA was then electroporated into *Y*. *pestis* KIM6+ expressing recombinase via plasmid pKOBEG-sacB. After growth on kanamycin plates, several colonies were tested for the correct Δ*rfaH* mutation by PCR (Supplementary Fig. S1). An allelic exchange plasmid pRE112^42^ was used to create the mutation in *Yptb*. The *rfaH* upstream and downstream region from the *Y*. *pestis* Δ*rfaH* mutant was amplified by PCR and ligated into pRE112 using the SacI and KpnI restriction sites, and transformed into chemically competent *E*. *coli* MFDλpir^43^. The resulting suicide plasmid was transferred in bi-parental matings with *Yptb*, and transconjugants were selected by plating on media containing kanamycin and 10% sucrose. The desired mutation was verified by PCR (Supplementary Fig. S1).

To complement the mutant strains, the *rfaH* gene and its native promoter from either *Yptb* or *Y*. *pestis* were inserted into the SalI and XbaI sites of plasmid pACYC184^44^. The resulting plasmids (*rfaH*+ ) were then electroporated into the Δ*rfaH* mutant strains. A complemented strain was also constructed using transposon Tn7. The *rfaH* gene was inserted into the pGRG36 plasmid^45^, and electroporated into the *Yptb* Δ*rfaH* and *Ypestis* Δ*rfaH* strains. The Tn7 transposon inserts transgenes into a defined neutral site in the chromosome (*attTn7*). The insertions were verified using *attTn7* site primers.

### Lipopolysaccharide isolation and analysis

Bacteria were grown overnight at 21 °C or 37 °C in TB and adjusted to an A_600nm_ of 1.0. LPS was then extracted as described previously^18, 46^. Briefly, 1.5 ml cultures were pelleted and suspended in 200 µl of SDS sample buffer. The lysed cells were boiled for 15 min, cooled, and treated with proteinase K at 59 °C overnight. The samples were then extracted with Tris-saturated phenol at 65 °C for 15 minutes, and then with diethyl ether at room temperature. Following centrifugation, the bottom blue layer was collected which contained the isolated LPS. The extracted LPS samples were separated on 4–20% polyacrylamide gradient gels. The gels were stained using the Pro-Q Emerald 300 Staining kit (Invitrogen) following the manufacturer’s protocol.

### RNA Isolation and Gene Expression Analysis

RNA was isolated from cultures (n = 3 for wild type or Δ*rfaH* mutant strains) grown in TB at 37 °C for 6 hours using the rBAC RNA Isolation Kit (IBI Scientific) according to the manufacturer’s instructions. Residual DNA contamination was removed using the Ambion Turbo DNAse Free Kit (ThermoFisher Scientific) and the integrity of the isolated RNA was checked by agarose gel electrophoresis. The concentration of RNA was measured using a Nanodrop spectrophotometer, and cDNA was made from the RNA using a ProtoScript II First Strand Synthesis Kit (New England Biolabs). The cDNA samples were diluted to 70 ng/µL and used as template in quantitative PCR reactions (qPCR).

Primers specific for each gene were designed to give 100–150 bp products (Supplementary Table S1). Reactions consisted of qPCR 2× SybrGreen Master Mix, High ROX (Genesee Scientific) with 3 µM each of forward and reverse primers and were run on a StepOne Real-Time PCR System. The cycling conditions were the following: 95 °C for 15 min followed by 40 cycles of 95 °C for 15 seconds then 60 °C for 1 min. A melt curve analysis was then performed to confirm the specificity of the PCR amplification. The resulting Ct values were normalized to the stably-expressed gene *dnaE* ^47, 48^. Comparative ΔΔCt values were used to calculate the fold changes^49^. A one-sample T-test using GraphPad Prism software was performed to determine if the mean fold change for each gene was significantly different from a hypothetical value of 1.0 (no change).

### Antimicrobial chemokine binding assay

Bacterial binding to the antimicrobial protein CCL28 was measured as previously described^18^. Briefly, bacteria grown to mid-logarithmic phase at 37 °C were diluted in filtered PBS supplemented with bovine serum albumin (BSA). The bacteria were incubated with 250 nM Human CCL28, washed in PBS, and then incubated with biotin- conjugated anti-chemokine antibody. The percentage of bacteria with detectable CCL28 bound to the surface was then measured with fluorescent streptavidin conjugates using a BD Accuri C6 Flow Cytometer and analysed using FACSDiva software (BD Biosciences). The statistical significance of specific comparisons was assessed via two-way ANOVA with Dunnett’s correction using GraphPad Prism software.

### Antimicrobial peptide susceptibility assays

The ability of CCL28 or polymyxin to kill bacteria was tested as previously described^18^. Bacteria were diluted into 0.1 μM potassium phosphate buffer (PPB) and incubated with 250 nM CCL28 or 10 µg/µL polymyxin B diluted in 0.01 mg/ml BSA, or in BSA alone for 2 hours. Samples of the bacteria were removed and mixed with freshly prepared Polystyrene 15 μm Microsphere counting beads and Propidium Iodide (PI). The numbers of bacteria per 30,000 beads were then determined by flow cytometry as described above for the binding assay, and percent survival was calculated by dividing bacteria in the treated sample by the bacteria in the BSA control sample. The statistical significance of specific comparisons was assessed via two-way ANOVA with Dunnett’s correction using GraphPad Prism software.

### Mouse Infections and Ethics Statement

Mouse infections were carried out in a biosafety level 2 laboratory in accordance with standard operating procedures approved by the Brigham Young University Institutional Animal Care and Use Committee. Carriage of the pYV virulence plasmid in *Yptb* strain IP32953 colonies was assessed by growth on media containing Congo-red^20^ and verified by PCR targeting the *yopM* and *lcrV* genes. Wild-type and Δ*rfaH Yptb* bacteria were grown overnight at 28 °C in TB and then subcultured until they reached an A_600nm_ of 1.0. After centrifugation and resuspension in PBS, 100 µl of bacterial suspension was inoculated orally using a tube and ball syringe to BALB/c mice approximately 3 months of age. The numbers of bacteria in the inoculum was measured by serial dilutions and plating onto Yersinia Selective Agar (YSA) plates (which contain bile salts, crystal violet, and irgasan). For the P^+^ strains, mice were infected with 2 × 10^7^ CFU, and 2 × 10^8^ CFU were used for the P^−^ strains. The mice were fasted for 16 hours before infection and were then given food 2 hours following infection. After 3 days, the mice were sacrificed and the mesenteric lymph nodes, Peyer’s patches, spleens, and livers were collected. These organs were weighed, homogenized in PBS, and serial dilutions were plated onto YSA. Colonies were counted after 24 h of growth and the CFU/g of each organ was calculated. For intravenous infections, mice were infected retroorbitally under anesthesia with 100 µl of bacterial suspension containing 1 × 10^5^ CFU of P^−^ *Yptb*. After 3 days, the mice were sacrificed and the liver and spleen were harvested, homogenized, and then plated on YSA.

### Data Availability

Most of the data generated or analyzed during this study are included in this published article (and its Supplementary Information file). Other data are available from the corresponding author on reasonable request.

## Electronic supplementary material

Supplementary Information
