# Supplementary material for: Differential impact of lipopolysaccharide defects caused by loss of RfaH in Yersinia pseudotuberculosis and Yersinia pestis
Source: Sci Rep. 2017 Sep 7;7:10915. doi: 10.1038/s41598-017-11334-6 (PMC5589760; doi:10.1038/s41598-017-11334-6)
Supplement: Supplementary file 1 — Supplementary Information [file 41598_2017_11334_MOESM1_ESM.pdf]

SUPPLEMENTARY INFORMATION

Differential impact of lipopolysaccharide defects caused by loss of RfaH in *Yersinia pseudotuberculosis* and *Yersinia pestis*

Jared M. Hoffman[1], Shea Sullivan, Erin Wu, Eric Wilson, David L. Erickson

Department of Microbiology and Molecular Biology, 4007 LSB, Brigham Young University, Provo UT 84602

[1]current address: Utah Public Health Laboratory, 4431 South 2700 West, Taylorsville, UT 84129

Corresponding author: david_erickson@byu.edu

Supplementary Table S1. Primers used in this study.

Primer name	Sequence (5’ - 3’)	Purpose
rfaH 500 F SacI	GCCGAGCTCGCCAATAATTTACCAACATCTCGC	*rfaH* gene deletion
rfaH 500 R KpnI	CCGGGTACCGCTAAGTATGCGTCTTGCTACTATG	*rfaH* gene deletion
rfaH F	GCCGCGTCGACTTCGGCGGCTATGGGATGCG	*rfaH* gene deletion and complementation
rfaH R	CCGCCTCTAGAGTGCAGGTGCGGATGGCGTA	*rfaH* gene deletion and complementation
rfaH internal F	GGCTCAAGCTTCCCTTAGGACACCTTCATGGAC	*rfaH* gene deletion
rfaH internal R	GCGTGTCTAGAGGCGTTTAGTTTTCGTTAACTTATCTGGGC	*rfaH* gene deletion
lcrV F	TCGCCGAATACACAATGGGA	Virulence plasmid verification
lcrV R	GAGCAGGTGGTGGCAAAGT	Virulence plasmid verification
yopM F	CGACAAGCCCATGAGCTAGA	Virulence plasmid verification
yopM R	GATTCCAGGCTCTGCGGTAA	Virulence plasmid verification
attTn7 F	CGCTAACAACCCTGCGACTTCA	Tn7 insertion verification
attTn7 R	GCGTGCTCGTGGTGGTTTGC	Tn7 insertion verification
ddhD F	TATTGGGGAATGCCAGCAGG	qPCR
ddhD R	TGGCTCCGGTCCAAGTACTA	qPCR
ddhA F	TCTACTTGGGAGCAGGAACC	qPCR
ddhA R	CACGCAACGTATCCATTGGC	qPCR
ddhB F	CGATGCTGAGGCTACTCCAGG	qPCR
ddhB R	ATGAGGGTGAGCATTGCCAT	qPCR
wbyI F	GCGGTACACTCGGAGTTGTA	qPCR
wbyI R	TCCAAAAATCATAATCACTGACAGGA	qPCR
wbyK F	TGCGCGTGGTGGTTATAAGA	qPCR
wbyK R	ACTAGTGAATGCCCCTCCAC	qPCR
waaC F	CGTGTGGTACAACAACGCAG	qPCR
waaC R	ACTTTTACAATCCGGCCCGT	qPCR
waaE F	AACGGCACACGCTACTCAAT	qPCR
waaE R	CTCGATATCATGCGGCCCAT	qPCR
waaQ F	CAGCGTCATTGTCCCCGTAT	qPCR
waaQ R	AGAGCCATCGTTAACGGCAA	qPCR
wabD F	TGGCCACATTATGCCTGGT	qPCR
wabD R	TTGGGCCACGAGTGCTAATT	qPCR
wabC F	GAGCCACAATTGCAGGCAAA	qPCR
wabC R	ACTTATCCCAACTCCAGCGC	qPCR
dnaE F	TTTCGTCCCGGTCCGTTACAA	qPCR
dnaE R	ACAAGATTATGCCGTAGGTCG	qPCR

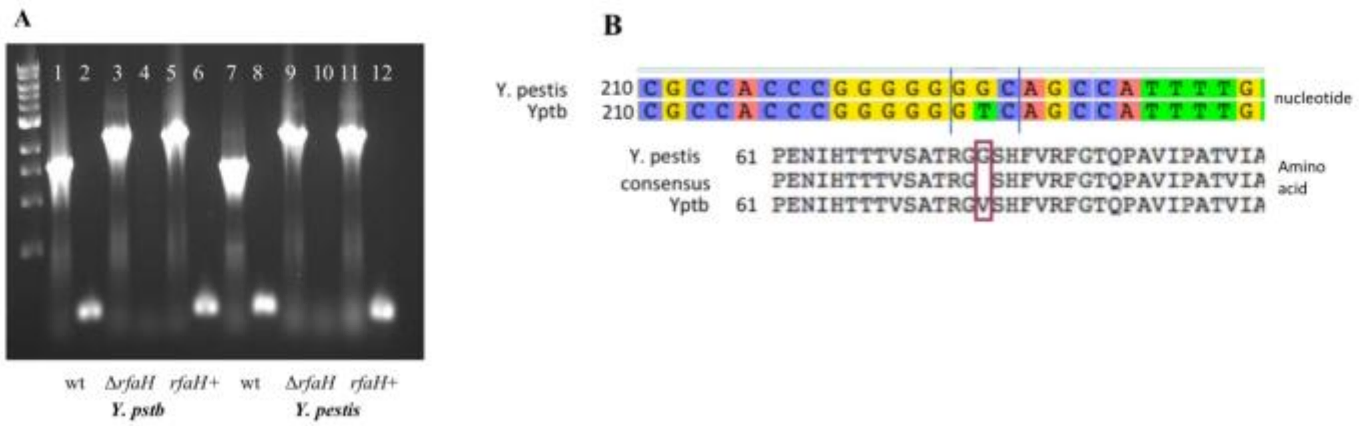

Supplementary Figure S1. Deletion and complementation of *rfaH* in *Yptb* and *Y. pestis*. A. Agarose gel electrophoresis of PCR reactions. Reactions were performed using primers flanking the *rfaH* gene (500 F & R primers) to show an increased product size in Δ*rfaH* mutant (3, 9) and *rfaH*+ complemented strains (5, 11) in both *Y. pstb* and *Y. pestis.* The wild-type strains (1 and 7) gave a shorter product indicating the Km^R gene (which is larger than the *rfaH* gene) was inserted. Lanes 2, 6, 8, 12 were performed using the *rfaH* internal primers (150 bp product) which show *rfaH* gene present in wild-type and *rfaH*+ complemented strains, but not Δ*rfaH* (Lanes 4 and 10). **B.** Comparison between the *rfaH* gene in *Y. pestis* (top) and *Yptb* (bottom). They share a 99.8% identify with only one G-T nucleotide difference at position 224, resulting in a predicted Glycine-Valine difference at amino acid 75. Plasmids containing either the *Yptb* or *Y. pestis* versions of the *rfaH* gene were created for complementation analysis, and both gave identical results.
